# NTRC and TRX-f Coordinately Affect the Levels of Enzymes of Chlorophyll Biosynthesis in a Light-Dependent Manner

**DOI:** 10.3390/cells12121670

**Published:** 2023-06-20

**Authors:** Daniel Wittmann, Peter Geigenberger, Bernhard Grimm

**Affiliations:** 1 Institute of Biology/Plant Physiology, Humboldt-Universität zu Berlin, 10115 Berlin, Germany; wittmada@hu-berlin.de; 2 Department Biology I, Ludwig-Maximilians-University Munich, 82152 Planegg-Martinsried, Germany

**Keywords:** thioredoxin, NADPH-dependent thioredoxin reductase, chlorophyll synthesis, 5-aminolevulinic acid synthesis, chloroplast biogenesis, photosynthesis

## Abstract

Redox regulation of plastid gene expression and different metabolic pathways promotes many activities of redox-sensitive proteins. We address the question of how the plastid redox state and the contributing reducing enzymes control the enzymes of tetrapyrrole biosynthesis (TBS). In higher plants, this metabolic pathway serves to produce chlorophyll and heme, among other essential end products. Because of the strictly light-dependent synthesis of chlorophyll, tight control of TBS requires a diurnal balanced supply of the precursor 5-aminolevulinic acid (ALA) to prevent the accumulation of photoreactive metabolic intermediates in darkness. We report on some TBS enzymes that accumulate in a light intensity-dependent manner, and their contents decrease under oxidizing conditions of darkness, low light conditions, or in the absence of NADPH-dependent thioredoxin reductase (NTRC) and thioredoxin f1 (TRX-f1). Analysis of single and double *trxf1* and *ntrc* mutants revealed a decreased content of the early TBS enzymes glutamyl-tRNA reductase (GluTR) and 5-aminolevulinic acid dehydratase (ALAD) instead of an exclusive decrease in enzyme activity. This effect was dependent on light conditions and strongly attenuated after transfer to high light intensities. Thus, it is suggested that a deficiency of plastid-localized thiol-redox transmitters leads to enhanced degradation of TBS enzymes rather than being directly caused by lower catalytic activity. The effects of the proteolytic activity of the Clp protease on TBS enzymes were studied by using Clp subunit-deficient mutants. The simultaneous lack of TRX and Clp activities in double mutants confirms the Clp-induced degradation of some TBS proteins in the absence of reductive activity of TRXs. In addition, we verified previous observations that decreased chlorophyll and heme levels in *ntrc* could be reverted to WT levels in the *ntrc/Δ2cp* triple mutant. The decreased synthesis of 5-aminolevulinic acid and porphobilinogen in *ntrc* was completely restored in *ntrc/Δ2cp* and correlated with WT-like levels of GluTR, ALAD, and other TBS proteins.

## 1. Introduction

Plants are exposed to the diurnal light–night rhythm, which is one of the fundamental exogenous regularities in nature. As sessile organisms, plants have adapted to the essential part of their metabolism to immediate, mostly post-translational responses to the constantly fluctuating light conditions and regularly changing environmental stimuli. This competence enables plants to have an optimized use of light as a vital energy resource and adaptability and viability during environmental changes. When photosynthesis evolved, protective mechanisms against excessive light excitation and a direct response to the transition from light to dark became crucial to prevent photooxidative damage. During excessive light intensities, the overexcitation of the light-harvesting complexes, the overreduction in the photosynthetic electron transport chain, and the excessive chlorophyll synthesis with accumulating free chlorophyll and tetrapyrrole intermediates have to be prevented [1]. 

During photosynthesis, electrons are transferred to the final electron acceptors, such as NADPH and thioredoxins (TRX) [2]. TRXs are small oxidoreductases that transfer the received electrons to redox-sensitive disulfide bonds between two cysteine residues of target proteins to reduce them to free thiol groups. The conformational changes in the tertiary and quaternary structure of the target proteins are induced by the opening of the disulfide bonds, which affects the activity or stability of the proteins [3,4]. 

Chloroplasts experience a strong change in reducing and oxidizing conditions during the daily photoperiodic cycles. Due to the diurnally changing photosynthetic activities, the chloroplasts are extremely exposed to varying redox conditions in light and darkness. It was observed that the reduced form of the redox-regulated proteins is usually the active form [5].

TRXs were first described in *E. coli* [6] and later in numerous other organisms. In heterotrophic cell compartments and organisms, TRXs are exclusively activated via NADPH-dependent thioredoxin reductases (NTR) [2,7]. The TRXs in photoautotrophic plastids differ from non-photosynthetic compartments of the cell. Plastidic TRXs are light-dependently reduced via ferredoxin:TRX reductase (FTR), and thereby act independently from the reduction of NADP^+^ [2,7].

Nevertheless, a C-type NTR (NTRC) also functions in chloroplasts [8] and, similar to TRX, can also reduce target proteins [9,10,11]. While plants lacking NTRC are viable, the knockout of FTR is lethal [12], emphasizing the importance of the TRX system for the development and maintenance of the metabolism. 

Half of the 20 TRXs found in Arabidopsis and several TRX-like proteins are localized in the chloroplasts [4]. The plastid-localized TRXs can be subdivided into five groups: f-type TRX (f1-2) with two, m-type (m1-4) with four, y-type (y1-2) with two isoforms, and x- and z-type TRX with one representative each [4]. Several potential plastid-localized target proteins of redox control were identified in in vitro and in vivo interaction screens [13,14,15]. So far, the interaction of TRX to its redox-controlled target proteins and the regulatory impact of a few redox-controlled proteins have been analyzed in detail [16]. Some of the redox targets belong to metabolic processes, such as the Calvin–Benson Cycle (CBC) [17], starch synthesis [18], or tetrapyrrole biosynthesis (TBS) [17,19], but transcriptional [20,21] and translational regulation [22,23], antioxidant defense [16], and signaling in plant immune responses [24] are controlled by TRX-mediated redox activities. 

Some enzymes of TBS have already been reported as redox-controlled proteins and interact with TRX and NTRC. The first redox-active cysteine residues of TBS enzymes were determined for the magnesium chelatase (MgCh) subunit CHLI and the Mg-protoporphyrin IX methyltransferase (CHLM) [25,26]. TRX- and NTRC-deficient mutants exhibited lower chlorophyll, and heme contents reflecting impaired TBS. Simultaneous knockouts of several TRX isoforms always have a stronger phenotypic impact on TBS and plant pigment content. The protein stabilities of various TBS enzymes, such as glutamyl-tRNA reductase (GluTR), glutamate-1-semialdehyde aminotransferase (GSAAT), 5-aminolevulinate dehydratase (ALAD), and CHLM or protochlorophyllide oxidoreductase (POR), were shown to be decreased to a different extent in NTRC, TRX-f, and TRX-m-deficient mutants [11,27,28]. Interestingly, the increased content of GluTR in TRX-m-deficient Arabidopsis plants is an exception [27]. 

Based on the additive negative effects on growth, chlorophyll content, and photosynthetic rates, as well as the lower activity of some redox-regulated enzymes in *ntrc/trxf1* [29] and *ntrc/trxf1/trxf2* mutants [30], a redundant functional overlap between the control mechanisms of FTR/TRX and NTRC has been suggested. However, recent evidence suggests that other TRXs, rather than NTRC directly, compensate for the absence of f-type TRXs in regulating CBC [30].

However, the absence of NTRC has, indirectly, a serious impact on the redox status of the entire TRX system due to its role as the primary electron donor of 2-cysteine peroxiredoxins (2-CPs). In the absence of NTRC, oxidized 2-CPs function as a sink for electrons from TRXs, leading to a tendency to oxidize and inactive TRX target enzymes [30]. The chlorotic *ntrc* phenotype was almost completely compensated by the simultaneous knockout of *NTRC* and *2-CPB*, combined with the knockdown of *2-CPA* (*ntrc/∆2cp*) [30]. It is proposed that TRXs normally provide compensatory electrons for oxidized 2-CPs in the *ntrc* background. The drastically decreased content of 2-CPs in the *ntrc/∆2cp* triple mutant corresponds to a lower need for electrons for 2-CP reduction, which would compensatorily allow sufficient use of the TRX-dependent reducing power to activate the target proteins [30]. The redox balance of 2-CPs is apparently important for the oxidative inactivation of reduced target enzymes in darkness [31,32,33]. In these studies, the oxidized 2-CP pool in darkness was identified as an electron sink of reduced target enzymes connected via oxidized TRXs or TRX-like proteins (reviewed by [16]).

This manuscript focuses on NTRC and the TRX-driven redox control of TBS enzymes. The aim of the presented studies was to investigate the combined impact of deficient TRX-f1 and NTRC on the stability of TBS enzymes under different light conditions. In continuation of previous reports on the effect of redox-sensitive CBC enzymes [30] and first TBS enzymes [34], we also examined whether the decreased enzyme stabilities and activities of TBS enzymes, such as GluTR and ALAD, can be restored in the NTRC-deficient mutants by additional downregulation of 2-CPs. Moreover, first hints are presented for the role of the Clp protease in the redox-dependent degradation of TBS enzymes by analyzing TBS protein levels after the inactivation of the *TRXm* genes in the *clpc1-1* background.

## 2. Methods

### 2.1. Growth Conditions

The *Arabidopsis thaliana* plants were grown on GS-90 standard soil mixed with vermiculite in a 3:1 ratio. Seeds placed on soil were stratified for 2 days at 4 °C and then grown under short-day conditions (SD; 8–10 h light, 21 °C) and different light intensities. The plants were grown at normal light (NL; 100–160 µmol photons m^−2^ s^−1^), high light (HL; 220–500 µmol photons m^−2^ s^−1^), or low light (LL; 20–40 µmol photons m^−2^ s^−1^). 

### 2.2. Pigment Extraction and HPLC

For pigment extraction, the homogenized leaf material was resuspended in basic acetone (80% acetone, 10% 0.2 M NH_4_OH, 10% _dd_H_2_O (*v*/*v*)). Pigment extraction was carried out, avoiding light exposure. Afterward, the pigment extracts were centrifuged (16,100× *g*, 4 °C, 10 min), the supernatants were collected, and an aliquot was loaded onto the HPLC columns. From the extracts obtained, chlorophyll a and b, the tetrapyrrole intermediates Mg-protoporphyrin IX (MgP), MgP monomethylester (MME), protochlorophyllide (PChlide), and chlorophyllide (Chlide) were quantitatively determined. The extraction of non-covalently bound heme was performed from the white pellet of the pigment extraction (see above) by incubation with acidic acetone (80% acetone, 4% HCl, 16% DMSO (*v*/*v*)) at room temperature. See [35] for the details. 

### 2.3. Protein Extraction, SDS-PAGE, and Immunodetection

The leaf material was homogenized in liquid nitrogen and resuspended in protein extraction buffer (2% SDS (*w*/*v*), 56 mM NaCO_3_, 12% sucrose (*w*/*v*), 2 mM EDTA, pH 8.0). The extracts were incubated at 70 °C for 20 min and then centrifuged at 16,100× *g* for 10 min. The supernatant was transferred to a new reaction tube, and the protein concentration was determined using the Pierce™ Bicinchoninic Acid (BCA) Protein Assay Kit (Thermo Fisher, Norcross, GA, USA). The samples were adjusted to 1–2 μg/μL and supplemented with DTT (final concentration 100 mM). The samples were boiled, and 15–20 μg total protein was loaded onto the SDS polyacrylamide gel. After immunoblot transfer and incubation of the nitrocellulose membranes with specific antibodies (Table 1), immunodetection of the proteins was performed by a CCD camera (ChemoStar Imager, Intas, Gujarat, India). The protein content was quantified by [36], https://lukemiller.org/index.php/2010/11/analyzing-gels-and-western-blots-with-image-j/ (accessed on 16 November 2021). The means and standard deviations were determined from three biological replicates at least. Significant changes in protein content compared to the WT were marked with asterisks in the figures (*, *p* ≤ 0.05 > 0.01 and **, *p* ≤ 0.01, *t*-test).

### 2.4. RNA Extraction, Reverse Transcription, and Quantitative Real-Time PCR

The method for extraction from plant leaf tissue was described by [44]. For cDNA synthesis, RNA was treated with DNaseI (Thermo Fisher, Norcross, GA, USA) and subsequently transferred to a RevertAid Reverse Transcriptase reaction mixture (Thermo Fisher, Norcross, GA, USA) with Oligo(dT)18-Primers according to the manufacturer’s protocol. qPCR was carried out in a real-time PCR detection system (CFX96-C1000, Bio-Rad, Hercules, CA, USA) with SYBR green dye (Bio-Rad Laboratories GmbH, D-85622 Feldkirchen, Germany). The qPCR primers are listed in Table S1. Relative gene expression was calculated using the 2^−ΔΔCt^ method [45].

### 2.5. ALAD Activity Assay

For the determination of ALAD activity from leaf extracts, leaf material was homogenized in liquid nitrogen and resuspended in extraction buffer (25 mM Tris-HCl (pH 8.2)). After centrifugation (16,100× *g*, 4 °C, 10 min), the supernatants were transferred to new reaction tubes. The protein content of the extracts was quantified using the Pierce™ BCA Protein Assay Kit (Thermo Fisher). The reaction was started by adding one volume of 2-fold reaction buffer (50 mM Tris-HCl (pH 8.2), 20 mM MgCl_2_, and 10 mM 5-aminovelulinic acid). The samples were incubated for 90 min at 37 °C with constant shaking (600 rpm), and the reaction was stopped by adding one volume of cold TCA (10%). After centrifugation (16,100× *g*, 4 °C, 10 min), the supernatants were mixed with 1 volume of Ehrlich’s reagent (12.7% perchloric acid (*v*/*v*), 76.4% glacial acid (*v*/*v*), 67 mM HgCl_2_, 1.82% para-dimethylaminobenzaldehyde (*w*/*v*), diluted with _dd_H_2_O to 100%). The samples were briefly centrifuged (2500× *g*, RT, 3 min), and the formation of porphobilinogen was photometrically quantified at 555 nm. For the calculation of the porphobilinogen concentration, the specific extinction coefficient (60,200 M^−1^cm^−1^) was used [46].

### 2.6. ALA Synthesis Capacity Measurement

Leaves from 4-week-old plants were covered with incubation buffer (50 mM Tris, 40 mM levulinic acid, pH 7.2) and incubated for 3–4 h under their respective light conditions. The samples were homogenized, resuspended in extraction buffer (20 mM KH_2_PO_4_, pH 6.8), and centrifuged (16,100× *g*, RT, 20 min). Then, 100 μL of ethyl acetoacetate (EAA) was added to 400 μL of the supernatant, and the samples were boiled for 10 min at 95 °C. A fraction of the remaining supernatant was used to determine the protein concentration by the BCA assay kit (Thermo Fisher). The boiled samples were briefly cooled on ice, and one volume of Ehrlich’s reagent (see ALAD activity assay) was added. The samples were incubated for 5 min at RT and subsequently centrifuged (2500× *g*, RT, 3 min). The absorption was measured photometrically at 553 nm. The ALA concentration was calculated using an ALA calibration line.

### 2.7. Virus-Induced Gene Silencing

The gene-encoded TRX-m1, -m2-, and -4 were downregulated via the VIGS approach [47,48,49]. For this purpose, the pTRV2 vector with the coding sequences of *TRXm2* and *TRXm4*, which also significantly downregulates *TRXm1*, was transformed into Col-0 WT and *clpc1-1* (SALK_014058) leaves according to [49].

## 3. Results

### 3.1. The Double Knockout of NTRC and TRX-f1 Uncovers an Additive Effect on TBS

**Figure 1:**
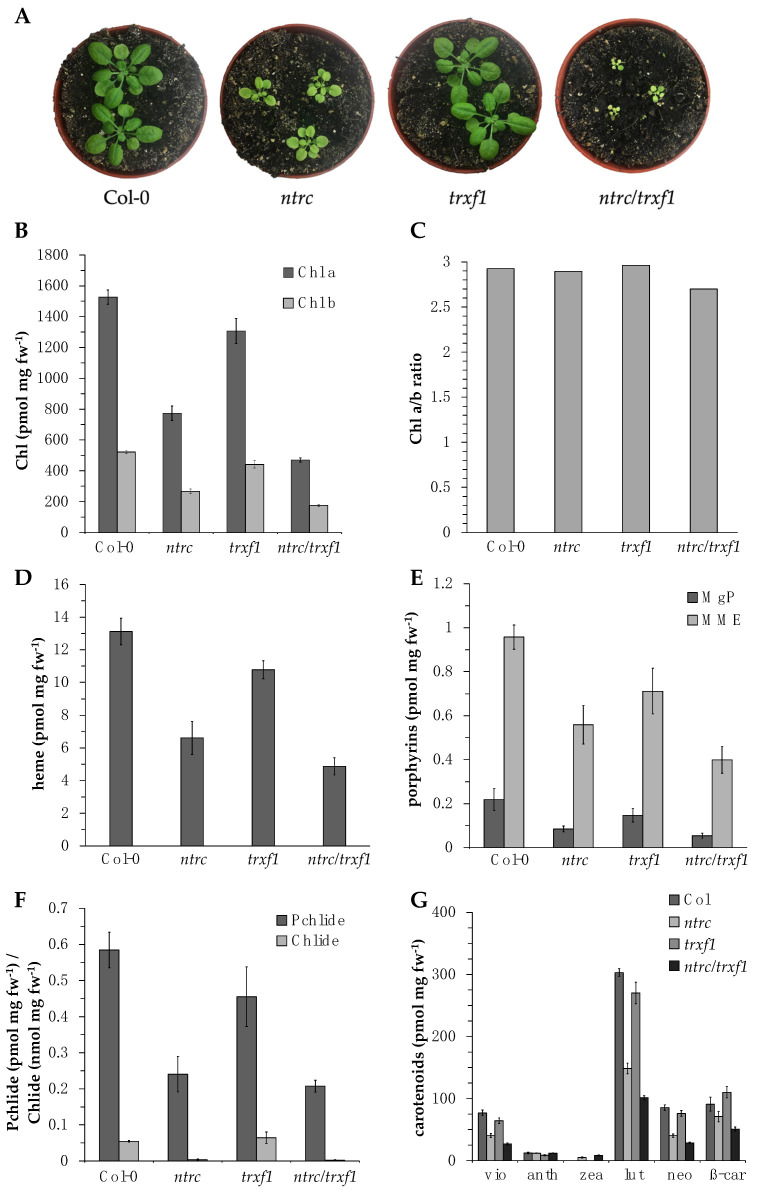
(**A**): Representative 28-day-old wild type (Col-0), *ntrc*, *trxf1,* and *ntrc/trxf1* seedlings grown under short-day (SD) conditions (10 h light, 14 h dark) and 100 μmol photons m^−2^ s^−1^. (**B**–**G**): analysis of TBS end-products and intermediates in *ntrc*, *trxf1,* and *ntrc/trxf1* compared to wild type. The pigments were extracted from the leaves of two-week-old seedlings grown under SD. (**B**): Chlorophyll content. (**C**): Chl a/b ratio. (**D**): non-covalently bound heme. (**E**): magnesium protoporphyrin IX (MgP) and magnesium protoporphyrin IX monomethyl ester (MME). (**F**): protochlorophyllide (Pchlide) and chlorophyllide (Chlide). (**G**): carotenoids violaxanthin (vio), antheraxanthin (anth), zeaxanthin (zea), lutein (lut), neoxanthin (neo), and β-carotene (β-car). Each result is the mean of at least three biological replicates.

The knockout mutant of the *Arabidopsis thaliana NTRC* gene (SALK_012208) [8,50,51,52] and the *TRX-f1* gene (SALK_128365) [29,53,54] have been well-characterized on multiple levels in previous studies. NTRC deficiency results in a growth-retarded, pale green mutant phenotype with 49% less chlorophyll compared to WT (Col-0, Figure 1A,B, Table 2, confirmed as previously described [8,50,51,52]). *trxf1* exhibits a 15% reduced chlorophyll content compared to WT plants but is phenotypically indistinguishable from WT (Figure 1A,B, Table 2). The *ntrc/trxf1* double mutant [29] shows a more remarkable decrease in chlorophyll content and slower growth than the two respective single mutants (Figure 1A). This observation confirms the additive effect of the missing two reductant genes [29]. The chlorophyll content of *ntrc/trxf1* diminished by 68% under SD conditions compared to WT. The chlorophyll a/b ratio in leaves hardly changes under the selected growth conditions in these three mutant genotypes compared to WT (2.9:1) and amounts to 3.0:1 (*trxf1*), 2.9:1 (*ntrc*), and 2.7:1 (*ntrc/trxf1*; Figure 1C). The accumulation of non-covalently bound heme and the steady-state levels of selected TBS intermediates are decreased to a relatively similar extent as chlorophyll levels in *ntrc*, *trxf1,* and *ntrc/trxf1* (Table 2, Figure 1D–G). We consider that the attenuated accumulation of tetrapyrrole end-products is generally the result of the compromised synthesis of tetrapyrroles and chlorophyll- and heme-binding proteins. Here, we focus on the effects of the absence of either or both of the reductants, NTRC and TRX-f1, on the overall TBS pathway.

### 3.2. Combined NTRC and TRX-f1 Deficiencies Lead to Lower Accumulation of Several TBS Enzymes in an Additive Manner

**Figure 2:**
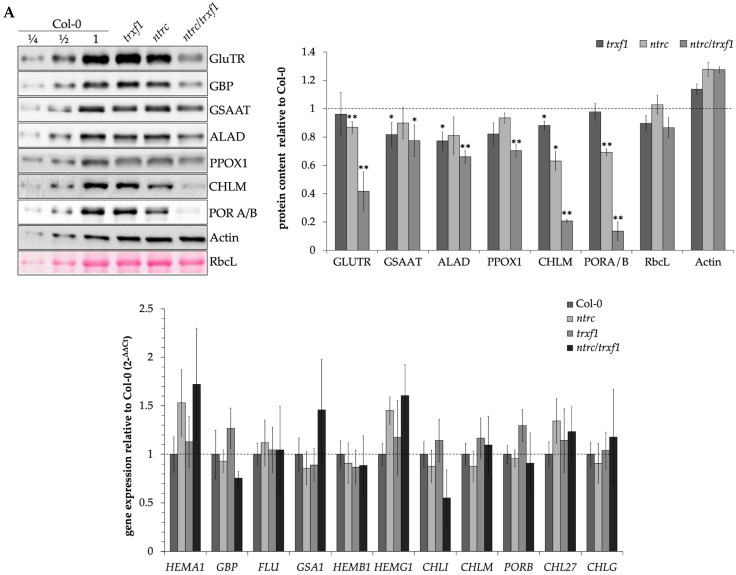
(**A**): The accumulation of selected TBS enzymes in *ntrc*, *trxf1,* and *ntrc/trxf1* compared to WT (Col-0). The protein samples were harvested from 14-day-old seedlings grown under SD conditions (120 μM photons m^−2^ s^−1^). (**B**): The protein content was quantified by determining the intensity of the immune-reacting protein band using ImageJ [36] and is displayed relative to the WT value. The means and standard deviations were determined from three biological replicates. Significant changes in protein content compared to WT were marked with asterisks in the figure (*, *p* ≤ 0.05 > 0.01 and **, *p* ≤ 0.01, *t*-test). (**C**): Determination of the relative transcript accumulation of different TBS genes in the mutants compared to WT by qPCR. The samples come from 14-day-old plants, which were grown in SD. The relative transcription levels compared to WT were calculated using the 2^−ΔΔCt^ method after normalization with *SAND* as the reference gene. Means and standard deviations refer to four biological replicates.

To compare the levels of TBS proteins in NTRC and TRX-f1-deficient seedlings with Col-0 WT, protein extracts of two-week-old seedlings were separated on SDS polyacrylamide gels and immunologically analyzed. Several TBS enzymes were less abundant in the *ntrc* mutant than the WT, confirming previous studies [11]. The accumulation of TBS enzymes was more drastically impaired in the *ntrc/trxf1* double mutant, while there were only minor effects in *trxf1* (Figure 2A,B). Compared to the WT, specifically, the contents of GluTR1, CHLM, and PORA/B decreased by 58%, 79%, and 86%, respectively, in *ntrc/trxf1*, but only by 13%, 37%, and 31%, respectively, in *ntrc*. Apart from these three most destabilized TBS enzymes in the double mutant, significantly diminished content (**, *p* ≤ 0.01, *t*-test) was determined for ALAD (34% less) and protoporphyrinogen oxidase (30% less), two other potential redox-dependent enzymes [55]. It should be noted that only slightly decreased contents for CHLM, GSAAT, and ALAD (12%, 18%, and 23%, respectively) were determined in *trxf1* compared to the WT (*, *p* ≤ 0.05, *t*-test). 

Quantitative real-time PCR (qPCR) was used to analyze whether the decreased content of TBS enzymes in the *ntrc* and *trxf1* mutants is caused by decreased transcriptional activity of the corresponding genes (Figure 2C). However, the results showed that the transcript levels were not decreased in response to the lower capacity of the plastid-localized reductants. The examined mRNA levels in the mutants were WT-like or slightly increased, except the decreased transcript contents of *GBP* (encodes the GluTR-binding protein) and *CHLI1* (encodes the CHLI subunit of MgCh) in *ntrc/trxf1*. These results indicate that the lower protein accumulation results from translational or post-translational modifications of the TBS proteins in response to a combined lack of NTRC and TRXf-1. Based on these results, it is concluded that the absence of TRX-f1 and NTRC affects the stability of the redox-controlled TBS enzymes GluTR1, GSAAT, ALAD, CHLM, and PORA/B in an additive manner. The sole lack of TRX-f1 has no obvious negative impact on the stability of GluTR and POR, which could be explained by the potential redundant activity of other plastid-localized TRX isoforms.

### 3.3. The Enzyme Activities of Early TBS Enzymes Are Decreased in Plants with an Additive Combined Effect of NTRC and TRX-f1 Deficiency

**Figure 3:**
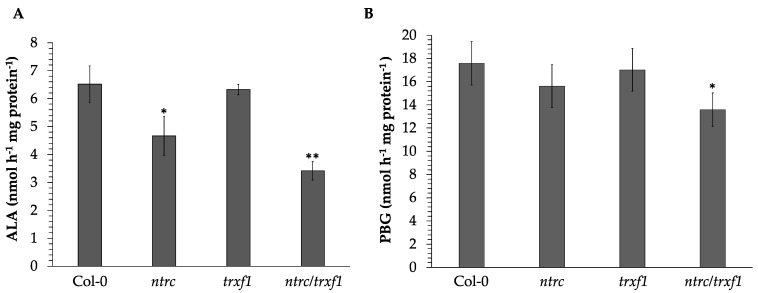
(**A**): ALA synthesis capacity of *ntrc*, *trxf1,* and *ntrc/trxf1* compared to WT(Col-0). The plants were grown for 4 weeks under SD (120 μmol photons m^−2^ s^−1^). (**B**): ALAD activity of soluble protein extracts from two-week-old SD-grown seedlings. Leaves were harvested at the end of the light period (after 10 h light). The results are given as means and standard deviations from at least three biological replicates. Significant changes from Col-0 enzyme activities were marked with asterisks in the figure (*, *p* ≤ 0.05 > 0.01 and **, *p* ≤ 0.01, *t*-test).

As shown previously, *ntrc* and *trxf1* single and double mutants showed decreased reduction states and activities of CBC enzymes, while their protein contents were not or only slightly decreased compared to the WT [29]. Therefore, we were interested in assessing the activities of TBS enzymes in these mutants. We assayed in planta the ALA synthesis rate, a combined assay of the enzyme capacity of GluTR and GSAAT, because there is no routine in vitro assay available for GluTR activity alone [56]. The ALA synthesis rate decreased by 28% in *ntrc* and 48% in *ntrc/trxf1* compared to the WT (Figure 3A). In contrast, *trxf1* had a WT-like ALA synthesis capacity (Figure 3A). The lower ALA synthesis rate correlates with the lower content of both TBS proteins in the mutants relative to the WT.

ALAD follows the ALA synthesizing enzymes in the TBS pathway and belongs to the redox-controlled enzymes [28,57]. The ALAD activity assay of light-exposed *ntrc* extracts showed an 11% decreased activity compared to the WT control; the ALAD activity in the double mutant was significantly decreased by 20%, while *trxf1* extracts contain WT-like activity (Figure 3B). The lower ALAD activity of the *ntrc* and *ntrc/trxf1* extracts correlates with the lower ALAD levels of these mutants. However, as a result of lower reducing power, ALAD activity is apparently less decreased than the accumulating ALAD contents in *ntrc* and *ntrc/trxf1* (Figure 2 and Figure 3). The decreased protein stability and activity of CHLM have already been reported for *ntrc* compared to WT plants [11]. These observations were confirmed. While the CHLM activity of *ntrc* decreased by 38% relative to the WT, the decline of its activity was more pronounced in the *ntrc/trxf1* double mutant (64% less compared to the WT).

### 3.4. High Light Leads to an Increase in GluTR Levels and Attenuates a Decrease in GluTR Content in Response to NTRC/TRXf-1 Deficiency

When the photosynthesis complexes in green leaves of adult plants are established, the need for new chlorophyll synthesis is mainly coupled to the turnover of chlorophyll-binding proteins of photosystem I and II (PSI and PSII). This turnover was mainly studied at the levels of the repair cycle of the D1 protein in PSII and the regular adjustment of the peripheral antenna complexes [58,59]. During high light (HL) stress, the reaction centers can be detrimentally excited, leading to over-reduced electron acceptors (Qa and the plastoquinone pool). When excess excitation energy cannot be dissipated via carotenoids to heat (NPQ), more ROS is formed at PSII [60]. In particular, the D1 subunit and attached pigments are susceptible to photooxidative damage. Consequently, HL induces an increase in D1 turnover and an increased demand for chlorophyll [61]. Consequently, increasing light intensities also trigger increased ALA synthesis capacity [62]. 

Thus, the redox-dependent levels of TBS enzymes should be examined at the end of the light phase, during the dark phase, and with increasing light intensity in *ntrc*, *trxf1,* and the double mutant. It was hypothesized that the photoperiodic growth of the NTRC/TRXf-1-deficient lines influenced the diurnal content of redox-dependent TBS proteins, and HL stress likely caused a shift between the reduced and oxidized forms of these enzymes, thus altering their redox-dependent stability.

As a result of photoperiodic growth under the short-day condition, *trxf1, ntrc,* and the double mutant had gradually decreased levels of GluTR, CHLM, and POR, and increased mutant phenotypes under both light and dark conditions. Since *ntrc* also contains less reduced TRXs in the stroma during the day than the WT [30], the lower reductive TRX pool could be a reason for the decreased content of these TBS proteins in the NTRC-deficient background. 

**Figure 4:**
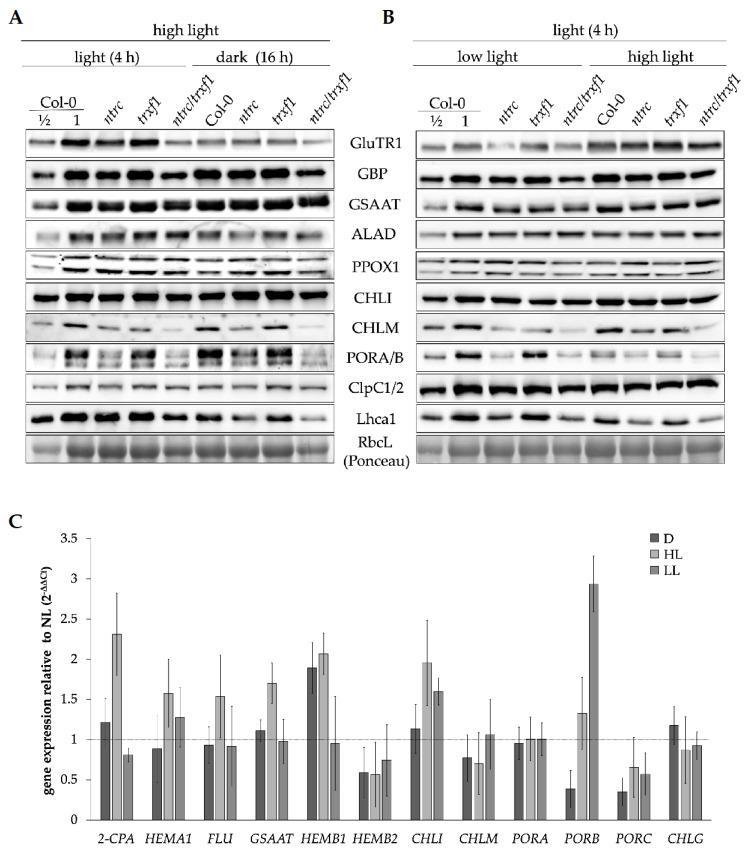
Immune analysis of TBS proteins and other plastid-localized proteins (as references) in light-exposed WT (Col-0), *ntrc*, *trxf1,* and *ntrc/trxf1* plants under different growth conditions. (**A**,**B**): The plants were initially grown for 3 weeks under SD (8 h light/16 h dark, normal light (NL, 160 µmol photons m^−2^ s^−1^). (**A**) Then, the SD-grown seedlings were exposed for an additional week to high light (HL 500 µmol photons m^−2^ s^−1^). Samples were harvested after 4 h of light and 16 h of darkness. (**B**) The SD-grown three-week-old seedlings were exposed to low (LL, 40 µmol photons m^−2^ s^−1^) or high light (HL) for a week and then, samples were harvested 4 h after the start of the light phase. (**C**): Expression of selected TBS genes in WT (Col-0) grown under different light conditions. SD-grown three-week-old plants under NL were either left in NL or transferred to HL or LL for another week. The gene expression of the different growth conditions is given relative to the expression under NL (dashed line), with *ACT2* (AT3G18780) as the reference gene. The leaf samples were harvested in the middle of the light period (4 h after the start of light exposure) or at the end of the dark period (16 h) of NL-exposed plants. The relative transcription was calculated using the 2^−ΔΔCt^ method. Means and standard deviations refer to four biological replicates.

Of the selected TBS proteins, GluTR1 content was most decreased at the end of the dark phase compared with the middle of the high light phase (HL, 500 µmol photons m^−2^ s^−1^) in all lines analyzed (WT, *ntrc*, *trxf1,* and *ntrc/trxf1,* Figure 4A). GluTR1 levels in *ntrc* and *ntrc/trxf1* changed between day and night but to a lesser extent compared to the WT and *trxf1* (Figure 4A). Apart from the varying GluTR accumulations during the day, only dark accumulation of ALAD was slightly lower than during light exposure of the plants (Figure 4A). Thus, ALAD might also be a candidate for light- and redox-dependent regulation of protein stability. The levels of other TBS enzymes, such as GSAAT, CHLM, and CHLI, did not vary between the light and dark phases. The use of an antiserum raised against the two POR isoforms A and B reveals that POR accumulates more strongly in the dark than in HL (Figure 4A).

In addition, the levels of the TBS enzymes were compared between HL and low light conditions (LL, 40 µmol photons m^−2^ s^−1^). Among the enzymes analyzed, only GluTR accumulation was more abundant under HL than LL (Figure 4B), while PORA/B was less abundant. The light-dependent increase in the GluTR content was observed in the WT and the three selected mutants. In *ntrc*, *trxf1,* and *ntrc/trxf1,* the effect on GluTR content was even stronger between HL- and LL-grown seedlings. This indicates that the effects of combined and single deficiencies of NTRC and TRXf-1 to decrease GluTR levels in LL are clearly attenuated under HL conditions.

Since the transcription of several genes encoded in TBS enzymes is also light-dependently regulated [63,64,65], the mRNA levels of the WT plants grown under different light intensities were examined by qPCR. Except for *HEMB1* (encoding ALAD1) and *PORB*, the TBS transcript levels of dark samples did not significantly differ from NL samples. In tendency, the HL-transcript levels of several TBS-related genes were higher than NL and LL samples, except for the *PORB* mRNA content. The observed slightly increased *HEMA1* gene expression in HL-exposed samples compared to NL/LL cannot explain the increased abundance of GluTR1 under HL conditions (Figure 4C). WT-*HEMB1* expression hardly differed between the dark phase and HL, but a transcript level that was two times higher was determined in comparison to NL/LL samples. However, these altered *HEMB1* transcript levels are unlikely to account for the difference in ALAD content in dark- and light-exposed samples.

### 3.5. Redox-Dependent Stability of TBS Proteins and the Importance of the Clp Protease for TBS Enzyme Degradation

Reduced content of several TBS proteins was determined in response to deficient *TRX* and/or *NTRC* expression compared to the WT as well as in LL- and dark-grown WT seedlings compared to HL-exposed seedlings (Figure 2 and Figure 4). We hypothesize that a lack of reducing power, as well as oxidizing conditions, promotes the degradation of redox-dependent TBS enzymes. One of the dominant plastidal proteases is the Clp protease. Therefore, we addressed the question of the extent to which Clp-dependent proteolysis of TBS proteins is a redox-regulated process. To investigate whether Clp-dependent degradation in TBS is controlled by TRX, we assayed the wild type and the *clpc1-1* mutants after virus-induced gene silencing (VIGS)-mediated inactivation of the three genes: *TRXm1, TRXm2,* and *TRXm4* [49]. A *GFP* silencing sequence was used as negative control control.

**Figure 5:**
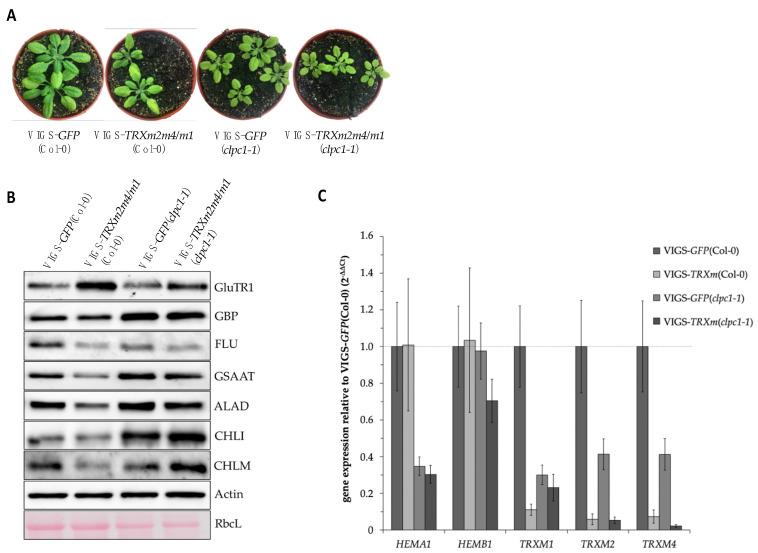
(**A**) Four-week-old representative plants that were grown under the long-day condition. (**B**) Western blot analysis of different TBS protein levels from VIGS-*TRXm2m4/m1*-(VIGS-*TRXm*) and VIGS-*GFP* plants in WT (Col-0) and *clpc1*-*1*. Plants were grown after infiltration for 3 weeks under long-day conditions at 120 μmol photons m^−2^ s^−1^. For the experiments, the light green leaves of the VIGS-*TRXm* plants were used. (**C**) Expression analysis of *HEMA1* and *HEMB1*, as well as the three silenced m-type *TRX* genes, via qPCR. The calculation of relative transcription was performed using the 2^−ΔΔCt^ method with *SAND* as the reference gene. Mean values and standard deviations were determined using three biological replicates.

The TBS proteins, which were less abundant in the *ntrc/trxf1* single and double mutants compared to the WT (Figure 2), accumulated to a lower extent in VIGS-*TRXm2m4/m1*(Col-0) relative to the control line (VIGS-*GFP*(Col-0)). In contrast, a higher amount of GluTR accumulated in TRX-m deficient leaves. In VIGS-*GFP*(*clpc1-1)* seedlings, GSAAT, ALAD, GBP, and CHLI accumulated more than the VIGS-*GFP*(Col-0) control. However, with the exception of CHLI, GluTR, and CHLM, the other TBS proteins had lower content under TRX-m deficiency in *clpc1*-*1* (VIGS-*TRXm2m4/m1*(*clpc1-1*, Figure 5a) compared to VIGS-*GFP*(*clpc1-1)*. The decreased protein levels of these enzymes resemble the protein instability in the *ntrc/trxf1* mutant. This finding suggests that the lower proteolysis of TBS proteins (enhanced stability of the TBS proteins) in *clpc1-1* is sometimes supported and sometimes compromised by TRX-m deficiency. In the absence of TRX-m isoforms, the accumulation of GSAAT, ALAD, and GBP decreased in *clpc1-1*, whereas the levels of GluTR, CHLI, and CHLM slightly increased compared to the VIGS-*GFP* control plants in the *clpc1-1* background. 

As an exception, VIGS-induced TRX-m deficiency caused a particular effect on GluTR content, when a more than twofold GluTR accumulation was observed in VIGS-*TRXm2m4/m1*(Col-0) compared to the VIGS-*GFP*(Col-0) control lines (Figure 5B and already presented in [27]). In contrast to the decreased GluTR content in *ntrc/trxf1* (Figure 2), the elevated GluTR accumulation in TRX-m-deficient plants is suggested to be due to a prevention of Clp-dependent degradation of GluTR1. This would result in an additional accumulation of GluTR in the VIGS-*TRXm2m4/m1*(Col-0) lines. However, a lower GluTR content was observed in the *clpc1-1* seedlings (with and without TRX-m deficiency relative to VIGS-TRX*m2m4/m1*(Col-0), which could be explained by the diminished *HEMA1* expression (Figure 5C; further explanation will be given in the discussion section). 

In general, it can be concluded—apart from a principled individual regulation of TBS enzymes as observed for GluTR—that the accumulation and stability of some analyzed TBS proteins, mainly in the early steps of TBS, in the *clpc1-1* mutant with reduced TRX activity point to a Clp system-induced post-translational control that has a redox-regulated effect on oxidized TBS proteins.

### 3.6. Influence of 2-Cysteine Peroxiredoxins on the Stability of TBS Enzymes

Our results above show that both NTRC and TRX-f thiol redox systems have overlapping functions and coordinately participate in the regulation of TBS protein levels. It has been shown previously that NTRC and TRX-f1 interact in an indirect manner via the redox balance of 2CPs. Pérez-Ruiz et al. (2017) [30] showed that the pale green, slow-growing phenotype of the *ntrc* mutant is suppressed by a simultaneous knockout of *2-CPB* and knockdown of *2-CPA* in a triple mutant (*ntrc/∆2cp*). The enzymes FBPase, phosphoribulokinase, and the f-type TRXs, which are mainly oxidized in *ntrc* even under light exposure, are predominantly reduced in *ntrc/∆2cp*, similar to the WT [30]. In a previous report, less abundant GluTR and CHLM contents of *ntrc* were shown to be recovered in *ntrc/∆2cp* compared to the WT [34], indicating that similar mechanisms are acting in the regulation of TBS enzymes. 

**Figure 6:**
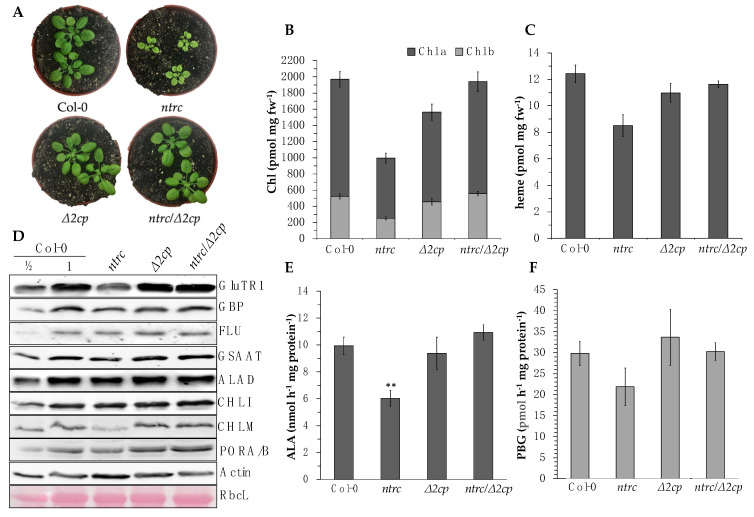
(**A**): Representative image of four-week-old WT (Col-0), *ntrc*, *∆2cp,* and *ntrc/∆2cp* plants grown under SD and SL conditions (120 µmol photons m^−2^ s^−1^). For the following experiments, the seedlings were harvested after 2 weeks. (**B**,**C**): Chlorophyll and heme contents of the mutants described in A compared to Col-0 analyzed by HPLC. (**D**): Western blot analysis to quantify selected TBS enzymes. (**E**): Determination of ALA synthesis capacity. The means and the standard deviations of four biological replicates per line are given. (**F**): Determination of the ALAD activity of the leaf extracts. The means and the standard deviations of three biological replicates are indicated. The statistical significance of the activity differences to the WT from E and F was determined using Student’s *t*-test and marked with an asterisk (**, *p* ≤ 0.01).

To verify this for other TBS proteins, their redox-dependently restored accumulation was analyzed in *ntrc/∆2cp* compared to the WT, *ntrc,* and *Δ2cp*. Careful quantification of protein content from three biological replicates is displayed and combined with enzyme activities for redox-controlled proteins. The phenotypical growth of *ntrc/∆2cp* was WT-like, and its chlorophyll and heme contents confirmed the recovery in comparison to *ntrc* (Figure 6A–C). As a confirmatory positive control for the redox-dependent protein accumulation [30,34], lower and WT-like CHLM values were indicated in *ntrc* and *ntrc/∆2cp*. Normalized to actin, the GluTR and GSAAT levels decreased in *ntrc* by 34% and 23%, respectively, compared to the WT, while in *ntrc/∆2cp,* the protein amounts increased by 19% and 16%, respectively, relative to the WT (Figure 6D). The ALAD content was 29% lower in *ntrc* relative to the WT (Figure 6D), while *∆2cp* and *ntrc/∆2cp* extracts showed an increased ALAD accumulation. The modified protein contents in the analyzed lines correspond to the ALA synthesis capacity and ALAD activity. While the ALA synthesis capacity was decreased by 39% in *ntrc*, the WT activity was restored in *ntrc/∆2cp* (Figure 6E). The ALAD activity decreased by 27% in *ntrc* and reached WT levels in *ntrc/∆2cp* (Figure 6F). It is obvious that the WT-like restored accumulation of redox-dependent TBS enzymes correlates with their activities in *ntrc/∆2cp,* which always results in a WT-like steady-state level of the TBS end-products chlorophyll and heme (Figure 6B,C).

## 4. Discussion

### 4.1. Different Concepts of Redox Control: Regulation of Protein Degradation vs. Regulation of Enzyme Activity

Except for the pale green *ntrc* [8] or the severe albino phenotype of the *trxz* mutant [20], a macroscopic phenotype of impaired seedling growth was only observed in the simultaneous inactivation or absence of more than one plastid-localized TRX isoform. Previous examples have been reported, such as the pale green leaf phenotype obtained by the VIGS of three different *TRXM* genes encoding the TRX-m isoforms [12,27] or the *trxf1/trxf2* mutant, whose growth is impaired under short-day conditions due to an impaired CBC activation [66]. In *ntrc/trxf1*, the redox state of redox-sensitive enzymes, such as FBPase, is shifted to an oxidized state, which clearly correlates with decreased enzyme activity [29]. 

In contrast to these observations, these TRX-deficient mutants display a different impact of the redox-dependent control on TBS enzymes. It is striking that no oxidized forms of TBS proteins were detectable in the TRX- and NTRC-deficient single and double mutants (Supplemental Figure S1). This is consistent with previous observations in *ntrc* [11], *ntrc/trxf1* [28], and the inducible VIGS*-TRXm2m4/m1* mutants [27]. It is obvious that the redox control of TBS enzymes does not primarily affect their activity, but their stability. 

While these observations point to functional redundancies of TRX isoforms and NTRC on TBS enzyme stability, they also highlight a different regulatory concept of these thiol-redox modulators on their target proteins in TBS compared to CBC and other described redox-regulated pathways. Firstly, the presented results about the content of TBS enzymes and their activities in *TRX-f1* and *NTRC* single and double knock-out mutants do not rule out the overlapping function of TRX-f1 and NTRC in the direct regulation of these enzymes involved in TBS metabolism. The common positive surface charge of the two f-type TRX isoforms and the TRXd from NTRC refers to similar substrate specificity [67]. This potential structural resemblance could explain the decreased accumulation of several TBS enzymes in *trxf1* and *ntrc* in comparison to the WT. 

These analyses also point to a different mode of action of the redox control. While the activities of the redox-sensitive CBC enzymes are regulated by their redox state [17], the redox control of TBS enzymes dominantly affects their stability, since combined deficiency of NTRC and TRX results in a redox-dependent proteolysis of these target proteins (Figure 2A and Figure 4A,B). This refers to a different concept of redox control, which does not directly determine the active state of TBS proteins but links the redox state of TBS enzymes with a pathway to protein degradation. Due to decreased TBS enzyme levels in the *trxf1* and *ntrc* mutants, it is also evident that lower enzyme activity, and consequently, lower chlorophyll and heme accumulation, are observed. Moreover, since the GSAAT content is also slightly decreased in the single and double TRX-f1- and NTRC-deficient mutants, additive effects of reductants on GluTR and GSAAT content and the ALA synthesis capacity could not be ruled out. The decreased ALA synthesis capacity and the ALAD activity in *ntrc* (Figure 3A) are additionally diminished in *ntrc/trxf1* (Figure 3A). Thus, it can be concluded that both, TRX variants and NTRC, control the turnover of enzymes of several TBS steps. 

The degradation of GluTR in darkness has been verifiably described at the end of the dark period, an observation that was linked to Clp-dependent degradation [68,69]. Dark degradation of ALAD was also observed but was not as pronounced as GluTR (Figure 4A, Supplemental Figure S2). The target proteins of the Clp protease are detected by the adaptor complex consisting of ClpS1 and ClpF [70,71]. Using affinity purification, GluTR and ALAD have already been identified as potential targets of ClpS1 [68,71], and GluTR was confirmed later as a Clp substrate [68]. To demonstrate how oxidizing conditions make TBS proteins more accessible to a redox-based proteolytic degradation and cause a redox-dependent proteolytic cleavage, VIGS of *TRXm2m4/m1* genes were silenced in WT and *clpc1-1* plants. We showed that several TBS proteins (GSAAT, ALAD, CHLI, GBP) accumulated in the *clpc1-1* mutants compared to the wild type. In the absence of TRX isoforms, such as the m-type TRX, GSAAT, ALAD, GBP, and FLU, the content was diminished (Figure 5B). Thus, the TRX isoforms and NTRC are proposed to be involved in the reduction in GSAT, ALAD, and some other TBS proteins, whose Clp protease-dependent degradation are prevented. 

Because the amount of ALAD, GSAAT, and other TBS enzymes decreased in *ntrc, trxf1,* and VIGS-*TRXm* seedlings, as well as in LL- and dark-grown plants compared to light-exposed plants (Figure 2, Figure 4 and Figure 5), it can be assumed that part of the pool of these TBS proteins can be oxidized, triggering a degradation signal for the Clp protease and other plastid proteases. Eventually, the oxidized state would lead to immediate protein degradation. Thus, the accumulation and stability of the analyzed TBS enzymes in the *clpc1-1* mutant could explain that the Clp-dependent proteolysis is a redox-regulated process.

The increased stability of GluTR1 in the VIGS-*TRXm2m4/m1* plants contrasts with the decreased accumulations of other TBS enzymes, such as GSAAT, ALAD, CHLI, and CHLM in TRX-deficient plants. When the oxidized state of the redox-controlled TBS proteins promotes their degradation, then we assume that GluTR remains in the reduced state in the TRX-m-deficient seedlings during light exposure (Figure 5). Therefore, we speculate that TRX-m could serve as an electron acceptor for GluTR, which could explain the higher GluTR accumulation in TRX-m-deficient plants (Figure 5) and its enhanced degradation during dark incubation. The TRX-m function for GluTR oxidation possibly occurs in concert with the regulation of GluTR via the GBP or the cpSRP43 interactions [59], which protect the protein from degradation and oligomerization/aggregation. However, the TRX-m activities require further investigations and are contrary to the role of TRX-f and NTRC, which are detectable in their single and double mutants, as well as in dark-grown seedlings, when the amount of GluTR is diminished compared to the light-exposed wild type. Then, the oxidized portion of GluTR would be increased, facilitating the proportional degradation of GluTR.

We propose that GluTR is one of the main targets of redox control. Decreased GluTR content in the mutants is likely responsible for the lower ALA synthesis rate, and, consequently, affects the accumulation of the metabolic intermediates and end-products. However, we have to keep in mind that not only the total amount of GluTR but also the subplastidal localization of GluTR determines the ALA synthesis rate. Previous reports emphasize that the soluble GluTR located in the stroma correlates with ALA synthesis [62]. It is currently not clarified whether the GluTR transition between the subplastidal compartments of stroma and membrane is also redox-controlled. However, with regard to the previous paragraph, the soluble Clp protease degrades GluTR, and it is suggested that the oxidized GluTR portion is recognized as a target of the Clp selector and the chaperone subunits. 

Finally, it cannot be excluded that the deficiency of parts of the thiol-redox system in plastids leads to modified plastid-derived retrograde signaling affecting the nuclear gene expression, or a decreased supply of the substrate glutamate or glutamyl-tRNA^Glu^ for ALA synthesis, since other metabolic processes in the chloroplasts of the mutants are also impaired. Due to the mainly oxidized TRXs, especially in *ntrc* [30], and the plastidal transcription (e.g., tRNA^Glu^) [20,21,72], the plastid-localized translation [22,73] and the protein import into plastids [74,75,76] are affected. 

### 4.2. TRXs Are Involved in the Regulation of Redox-Sensitive TBS Enzymes

Interestingly, the absence of 2-CPs rescues the NTRC-deficient phenotype to WT-like plants, which was also observed at the level of CBS enzymes and their activity [30]. The analyses of *ntrc/Δ2cp* confirm that the lower levels of GluTR and CHLM and the resulting lower accumulations of chlorophyll observed in *ntrc* leaves are remedied [34]. Here, we showed that the stability of GluTR and ALAD, and thus the corresponding rate of ALA and porphobilinogen synthesis, is mainly regulated by TRXs and, to a lesser extent, by NTRC (Figure 6D–F). Following the proposed model [30], ALA synthesis capacity and ALAD activity were restored to WT levels due to the WT-like contents of GluTR, GSAAT, and ALAD accumulations in *ntrc/∆2cp* (Figure 6D). These observations confirm the previous concept that the pool of oxidized TRX isoforms is the result of their reduced activity on 2-CP, which appears to be required in the absence of NTRC and thus is the main cause of the decreased chlorophyll and heme synthesis. This also would imply that NTRC itself is not directly involved in the regulation of TBS but acts on TBS by maintaining the reduced 2-CP pool. However, based on the observed direct interactions between NTRC and TBS enzymes, as well as the in vitro stimulation of CHLM and CHLI by NTRC [10,11], it is not completely excluded that NTRC may temporally have a direct reductive effect on these targets, e.g., in darkness, during stress, or at certain developmental stages when the effect of TRX is limited.

### 4.3. Is the Light-Dependent Fluctuation of GluTR1 and ALAD Content Caused by Redox-Switches?

Our results show that HL acclimation leads to increased GluTR protein levels, suggesting that chlorophyll biosynthesis is promoted when environmental light intensities are increased. Indeed, enhanced turnover of chlorophyll-containing photosynthetic proteins under these conditions requires increased chlorophyll synthesis [61]. Consequently, increasing light intensities also trigger increased ALA synthesis capacity [62]. Interestingly, the effect of NTRC/TRX-f1 deficiency to decrease GluTR protein levels is dependent on light intensity and is mainly found in LL conditions but is strongly attenuated during HL acclimation. Obviously, in *ntrc/trxf1,* GluTR is more stable in plants grown in HL than LL**.** These differences in the GluTR content between HL and LL could occur because the TBS enzymes in the NTRC- and TRX-f1-deficient mutants are proposed to be relatively more abundant in their reduced form in HL than LL (Figure 4B). This has already been observed for the redox status of the plastidial FBPase in *trxf1*. In normal light, FBPase was found to be mostly oxidized in *trxf1*, while it was mainly reduced in the WT. In HL, the FBPase in *trxf1* was mainly reduced, although not as completely as in the WT [66]. In confirmation of this, *trxf1* single and *ntrc/trxf1* double mutants were found to be more strongly inhibited in photosynthetic growth under LL compared to HL conditions [29].

However, the contents of TBS enzymes should always be related to the transcript levels of the corresponding genes. As previously described for GluTR (*HEMA* [68]), *HEMB* expression is also not considered to be the cause of the decreased ALAD levels in the dark compared to HL samples (Figure 4A). Thus, it is evident that proteolytic degradation is responsible for the decreased GluTR and ALAD accumulation during dark incubation. Regarding the ClpC subunit of the Clp protease, the protein levels between HL and dark, as well as between HL and LL, were unchanged in the WT and the mutants examined (Figure 4A,B). This suggests that the turnover of ClpC is not subject to a light-dependent change. However, it remains currently unclear whether the activity of the Clp protease or substrate availability is light- or redox-dependently regulated.

## 5. Conclusions

NTRC and TRX-f thiol redox systems have overlapping functions and coordinately participate in the regulation of TBS proteins and end-product synthesis. We summarize that not the activity but the stability of TBS enzymes, such as GluTR, ALAD, GSAAT, CHLM, and POR, is the main target for redox regulation in response to different light intensities. The redox-dependent control of TBS protein stability contrasts with the redox regulation of enzyme activity observed in other plastid metabolic pathways, such as the Calvin–Benson cycle. We hypothesize that TBS enzymes are sensitized to protein degradation by their oxidation mediated by redox control. Moreover, the redox-dependent compensation in *ntrc/Δ2cp* leading to WT-like accumulation of TBS enzymes indicates that TBS is controlled by FDX-TRXs and that NTRC is interacting in an indirect manner via the redox balance of 2-CPs. 

## Figures and Tables

**Table 1:** List of all antibodies used in this work.

Antibody	Dilution	Reference
Primary antibodies without HRP		
α-Actin	1:5000	Agrisera (AS13 2640)
α-ALAD1	1:2000	Wittmann et al. (2018) [28]
α-CHLI	1:5000	Luo et al. (2012) [37]
α-CHLM	1:2000	Alawady and Grimm (2005) [38]
α-ClpC	1:5000	Agrisera (AS01 001)
α-FLU^TPR^	1:500	Hou et al. (2019) [39]
α-GBP	1:2500	Czarnecki et al. (2011) [40]
α-GluTR	1:2500	Hedtke et al. (2007) [41]
α-GSAAT	1:2000	Grimm et al. (1989) [42]
α-PORA/B	1:2000	Agrisera (AS05 067)
α-PPOX1	1:1000	Lermontova et al. (1997) [43]
Secondary antibody with HRP		
α-rabbit	1:20,000	Sigma-Aldrich Chemie GmbH, Taufkirchen, Germany.

**Table 2:** Relative pigment- and steady-state levels of selected TBS intermediates in *ntrc, trxf1,* and *ntrc/trxf1* in comparison to wild type (WT, Col-0, data in %). Samples were harvested during light exposure. The absolute values of tetrapyrrole end-products and intermediates are given in Figure 1.

%	Chl a	Chl b	Chl a + b	Heme	MgP	MME	PChlide	Chlide
**Col-0**	100	100	100	100	100	100	100	100
***ntrc***	51	51	51	52	34	58	41	26
***trxf1***	86	85	85	85	60	74	78	92
***ntrc/trxf1***	31	33	32	38	22	42	35	10

## Data Availability

The data presented in this study are available on request from the corresponding author.

## Supplementary Materials

The following supporting information can be downloaded at https://www.mdpi.com/article/10.3390/cells12121670/s1, Figure S1: AMS labeling of total leaf protein extracts to determine the light-dependent redox state of selected TBS enzymes in the WT (Col-0), *ntrc*, *trxf1*, and *ntrc/trxf1* plants. The redox-sensitive CBC enzyme FBPase was used as a control. The seedlings were grown under SD conditions (100 µmol photons m^−2^ s^−1^) for 2 weeks. The samples were harvested after 30 min of incubation at higher light intensity (220 μmol photons m^−2^ s^−1^) before harvesting. The dark extracts were obtained from plants harvested at the end of the dark period (16 h of darkness). Total protein extracts were labeled with AMS (+), separated by non-reducing SDS-PAGE, and transferred to a nitrocellulose membrane. Detection was carried out using specific antibodies indicated on the right; Figure S2: Stability of early TBS enzymes after dark incubation of the WT (Col-0) seedlings. The seedlings were grown in soil for 2 weeks under SD conditions (120 μmol photons m^−2^ s^−1^). The initial sample (0 h) was harvested in the middle of the light phase (5 h light), and the seedlings were incubated for up to 48 h in darkness. The leaves were harvested at indicated time points under a green light. The qPCR primers are listed in Table S1.

## Abbreviations

2CP: two-cysteine peroxiredoxin; ALA, 5-aminolevulinic acid; ALAD, 5-aminolevulinic acid dehydratase; anth, antheraxanthin; β-car, β-carotene; CBC, Calvin–Benson Cycle; Chl, chlorophyll; Chl27, encoding a subunit of magnesium protoporphyrin IX monomethyl ester cyclase; CHLI, subunit of magnesium chelatase; Chlide, chlorophyllide; CHLG, Chl synthase; CHLM, Mg-protoporphyrin IX methyltransferase; Col(0), Columbia (0) ecotype of *Arabidopsis thaliana*; FBPase, fructose-1,6-bisphosphatase; FLU, fluoresence in blue; GBP, GluTR-binding protein; GFP, green fluorescence protein; FTR, ferredoxin:TRX reductase; GluTR, glutamyl-tRNA reductase; GSAAT, glutamate-1-semialdehyde aminotransferase; *HEMA*, encoding GluTR; *HEMB,* encoding ALAD; HL, high light; LL, low light; lut, lutein; MgCh, magnesium chelatase; MgP, magnesium protoporphyrin IX; MME, magnesium protoporphyrin IX monomethyl ester; NL, normal light; NPQ, nonchemical quenching; NTRC, NADPH-dependent thioredoxin reductase; neo, neoxanthin; Pchlide, protochlorophyllide; POR, protochlorophyllide oxidoreductase; PS, photosystem; qPCR, quantitative real-time PCR; ROS, reactive oxygen species; SD, short-day condition, SDS, sodium dodecylsulfate; TBS, tetrapyrrole biosynthesis; TRX, thioredoxin; VIGS, virus-induced gene silencing; vio, violaxanthin; WT, wild type; zea, zeaxanthin.
