# Supplementary material for: NTRC and TRX-f Coordinately Affect the Levels of Enzymes of Chlorophyll Biosynthesis in a Light-Dependent Manner
Source: Cells. 2023 Jun 20;12(12):1670. doi: 10.3390/cells12121670 (PMC10297434; doi:10.3390/cells12121670)
Supplement: Supplementary file 1 [file cells-12-01670-s001.zip › cells-2343807-supplementary.pdf]

Supplemental information

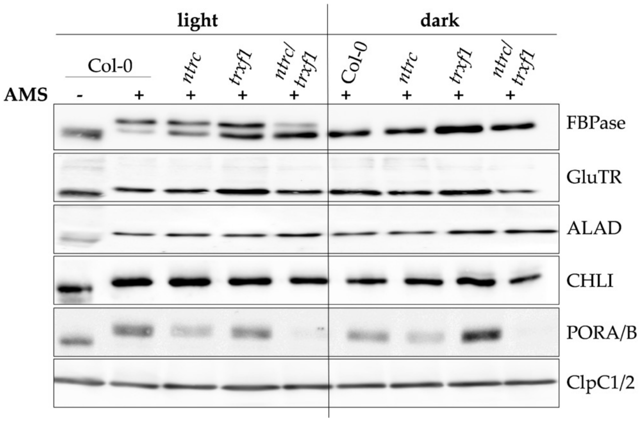

Figure S1: AMS labeling of total leaf protein extracts to determine the light-dependent redox state of selected TBS enzymes in WT (Col-0), *ntrc*, *trxf1*, and *ntrc/trxf1* plants. The redox-sensitive CBC enzyme FBPase was used as a control. The seedlings were grown under SD conditions (100 µmol photons m^{-2} s^{-1}) for 2 weeks. The samples were harvested after 30 min incubation at higher light intensity (220 µmol photons m^{-2} s^{-1}) before harvesting. The dark extracts were obtained from plants harvested at the end of the dark period (16 hours of darkness). Total protein extracts were labeled with AMS (+), separated by non-reducing SDS-PAGE and transferred to a nitrocellulose membrane. The detection was carried out using specific antibodies indicated on the right.

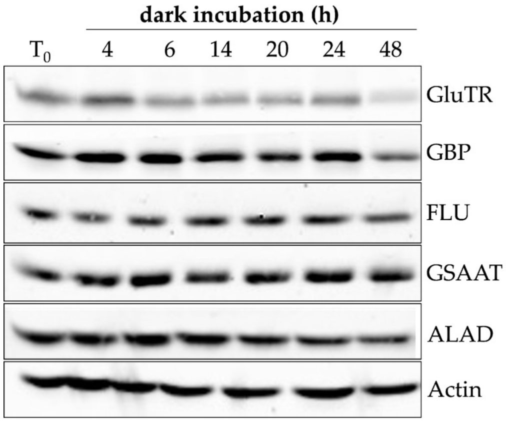

Figure S2: Stability of early TBS enzymes after dark incubation of WT (Col-0) seedlings. The seedlings were grown on soil for 2 weeks under SD conditions (120 µmol photons m^{-2} s^{-1}). The initial sample (0 h) was harvested in the middle of the light phase (5 h light) and the seedlings were incubated up to 48 h in darkness. The leaves were harvested at indicated timepoints under green light.
