# Supplementary material for: NTRC and TRX-f Coordinately Affect the Levels of Enzymes of Chlorophyll Biosynthesis in a Light-Dependent Manner
Source: Cells. 2023 Jun 20;12(12):1670. doi: 10.3390/cells12121670 (PMC10297434; doi:10.3390/cells12121670)
Supplement: Supplementary file 1 [file cells-12-01670-s001.zip › primer_table.pdf]

Name	Locus	Sequence
**TBS genes**		
HEMA1 fw<br>rev	AT1G58290	TTGCTGCCAACAAAGAAGAC<br>CCGTCTCCAATGAATCCCTC
GBP fw<br>rev	AT3G21200	TTCATGGAGGACGGAATCTG<br>TGTTAGCGTTAATCTGGTTGAC
FLU fw<br>rev	AT3G14110	AAGCCATACAGTATCACTCCA<br>TCCAGAATCTTCACTTTCCCT
GSA1 fw<br>rev	AT5G63570	TCAAAGAAGAGCGACACAGAG<br>GTAAACACCTTCTTCCAACATTCC
HEMB1 fw<br>rev	AT1G69740	AAGCTGCCGTTGCTGCTGGA<br>CGACGTGGGCGTCGGCTTAG
HEMB2 fw<br>rev	AT1G44318	AGGCATTGCTAGAAGCACGAGA<br>GGCGGATGCATAACAATGACTC
HEMG1 fw<br>rev	AT4G01690	GGGCCAAACAGTTGGTTCTT<br>GTTGTATCCTCCGCTCTCCA
CHLI1 fw<br>rev	AT4G18480	CGGTTATGAATGTAGCCACTG<br>CTTGCCCTACTATAGCTGC
CHLM fw<br>rev	AT4G25080	TTGCTGAAGCTGAGATGAAGGCAAAG<br>CAACGGTATCATACTTCCCAGTTAGG
CHL27 fw<br>rev	AT3G56940	GCTTCTTCTGCCTCTCGGTTTATG<br>GCCGTGGTTCGGTTTGTCTCG
PORA fw<br>rev	AT5G54190	TACCCTCTTCCCTCCTTTCC<br>GTTCCAGCTCCAATACACTCC
PORB fw<br>rev	AT4G27440	TGATTACCCTTCAAAGCGTCTCA<br>CAATGTATTCGTGTTCCCGGT
PORC fw<br>rev	AT1G03630	CTGGCAAAAGACTAGCACAGGTT<br>CAATACACTCCTGACTTCCCAAGAC
CHLG fw<br>rev	AT3G51820	TCATTCCTCAGATTGTGTTCCA<br>GTTACAAATATTCCGAGCACCA
**TRX family**		
TRXm1 fw<br>rev	AT1G03680	CATTTGGGACTCAAGAAG<br>GCTCGATTCCGGCAACAC
TRXm2 fw<br>rev	AT4G03520	TTCACTTGGGGATTTGGAGA<br>CCTGATGATTCCGGTAAGAC
TRXm4 fw<br>rev	AT3G15360	AAATTGTCACCCGTATCTG<br>TCCGCGAAGACTTCAAAC
**Reference genes**		
ACTIN2 fw<br>rev	AT3G18780	CCGGTATTGTGCTCGATTCTG<br>TTCCCGTTCTGCGGTAGTGG
SAND fw<br>rev	AT2G28390	AACTCTATGCAGCATTTGATCCACT<br>TGATTGCATATCTTTATCGCCATC
